# Protocol for the *Open Sky School*: a two-arm clustered randomized controlled trial to test the effectiveness of a nature-based intervention on mental health of elementary school children

**DOI:** 10.1186/s12889-023-15033-y

**Published:** 2023-02-03

**Authors:** Tianna Loose, Sylvana Côté, Catherine Malboeuf-Hurtubise, Jean-Philippe Ayotte Beaudet, Geneviève Lessard, Nicholas Chadi, Lise Gauvin, Isabelle Ouellet Morin, Marie-Claude Geoffroy

**Affiliations:** 1 Department of Social and Preventive Medicine, School of Public Health, University of Montréal, Montréal, QC Canada; 2 Sainte-Justine University Hospital Research Centre, Montreal, QC Canada; 3 Department of Psychology, Bishop’s University & CHU Sherbrooke Research Centre, Sherbrooke, QC Canada; 4 Department of Preschool and Elementary Education, University of Sherbrooke, Sherbrooke, QC Canada; 5 Department of Educational Sciences, University of Quebec in Outaouais, Gatineau, QC Canada; 6 Department of Pediatrics, University of Montreal, Montreal, QC Canada; 7 Department of Social and Preventive Medicine and Interdisciplinary Research Group On Health (GRIS), University of Montreal, Montreal, QC Canada; 8 School of Criminology, University of Montreal & Research Center of the Montreal Mental Health University Institute, Montréal, QC Canada; 9 Department of Educational and Counselling Psychology, McGill University, McGill University, Montreal, QC Canada; 10 Douglas Research Center and Department of Psychiatry, McGill University, Montreal, QC Canada

**Keywords:** School-based intervention, Mental health, Children, Randomized controlled trial, Nature, Outdoor education, Elementary-school children, Greenspace

## Abstract

**Background:**

This article outlines the protocol for a trial to test the effectiveness of a nature-based intervention called *Open Sky School* to reduce mental health problems among elementary school children. Experimental studies show that contact with nature (e.g. walks in parks) improve mental health. A growing number of teachers have been applying outdoor education within the regular school curriculum and evidence suggests that such teaching methods could improve students’ mental health but a randomized controlled trial has never been conducted.

**Methods:**

A two-arm clustered randomized controlled trial will be conducted in elementary schools across Québec, Canada. Following informed consent by teachers, parents and students, schools will be randomly assigned 1:1 to the intervention or the control group with a total of 2500 5-6^th^ grade students and 100 teachers expected to participate. The intervention will take place outdoors in a green-space (2 h per week for 12 weeks) and include a toolkit of 30 activities to foster well-being (e.g. mindfulness) and academic competencies (e.g. mathematics). Questionnaires will be administered to teachers and students before, immediately after and 3 months after the intervention. The primary outcome will be reductions of mental health problems in children from pre-to-post test (Social Behavior Questionnaire: self and teacher reports). Secondary outcomes include depression, positive and negative affect, nature connectedness, and pro-environmental behaviors among children. We will explore, immediate benefits on teacher’s well-being and positive and negative affect and sustained benefits among students at 3 months follow-up. For the primary outcome, we will explore moderators including child’s sex, child’s disability status, the green-space of neighbourhoods, the school’s socio-economic position and teacher’s experience.

**Discussion:**

In conducting the first randomized controlled trial of the *Open Sky School*, our results could provide empirical evidence on the effectiveness of nature-based interventions in reducing mental health problems among elementary school children.

**Trial registration:**

This study was registered with clinicaltrials.gov: NCT05662436 on December 22, 2022.

**Supplementary Information:**

The online version contains supplementary material available at 10.1186/s12889-023-15033-y.

## Background

Mounting evidence indicates that spending time in nature can be beneficial for mental health for individuals of all ages (e.g., to reduce symptoms of depression and anxiety) [1–4]. Indeed, large epidemiological studies have reported an association between residing in an area with a high density of green vegetation and mental health. For instance, a recent Danish study including over 900,000 children suggested that proximity to green space in childhood was associated with a lower onset of psychiatric disorders later in life, independently from other known risk factors such as the family socioeconomic status and parents’ psychopathology [5]. A double-blind, controlled, within-subject study carried out among children aged 7 to 12 years diagnosed with attention-deficit/hyperactivity disorder also suggested that walks lasting 20 min in urban parks reduced sustained attention difficulties more than walking in town or in the streets around their residence [6]. Large effect sizes were observed (0.52 and 0.77 respectively), which were comparable to the effect of psychostimulant medication. In addition to health benefits, spending time in nature during childhood is known to increase nature connectedness and pro-environmental attitudes and behaviors. This would be because when children spend time enjoying nature, they tend to feel more connected to the environment and become more likely to engage in behaviors to protect it as a result e.g. recycling, volunteering for pro-environmental causes [7]. As such, increasing time spent in nature at school could be a highly accessible way cultivate children’s pro-environmental attitudes and behaviors while also reducing mental health problems. Accessible interventions capable of reducing mental health problems in children are also particularly timely given numerous reports suggesting worsening levels of mental health among youth since the beginning of the COVID-19 pandemic [8] and increasing preoccupations for climate change among youth [9]. While the practice of outdoor education (e.g. teaching the standard curriculum off campus) has been increasingly encouraged in education, including in Canada, where a rising number of teachers practice outdoor education [10], the effectiveness of such practices in reducing mental health problems has never been tested with a robust randomized-control trial design.

We are nevertheless aware of two quasi-experimental studies that tested the effectiveness of education outside the classroom applied directly within the school curriculum in reducing mental health problems among students. The first study was carried out in Sweden with children aged 6 to 12 years (*n* = 230) and used a non-equivalent quasi-experimental design to assess whether daily teaching outside (e.g., in the schoolyard) for 6 months reduced symptoms of mental disorders, in comparison with usual indoor education. The intervention entailed one hour per day of outdoor education where children were taught academic competencies while being outside and manipulating elements of nature (e.g., branches and stones used to represent mathematical concepts rather than textbooks). Findings suggested a significant reduction in internalized and externalized symptoms pre-to-post intervention, but only among boys [11]. A second quasi-experimental study was conducted over the course of a school year with students aged 9 to 11 years (*n* = 631) and aimed in part to assess the benefits of outdoor teaching on mental health, in comparison to education as usual. The authors found that pro-social behaviors improved among all children and that hyperactivity and problems with peers were reduced among disadvantaged children [12]. However, quasi-experimental designs do not use randomization and conclusions about causal associations between the intervention and outcomes are limited. Further, the two quasi-experimental studies described above did not require that the outdoor education take place in a green space. Therefore, the effectiveness of education outside of the classroom that necessarily includes the component of exposure to nature in reducing mental health problems needs further investigation. In light of the available empirical evidence and the aforementioned limitations, we propose to test the effectiveness of a nature-based intervention implemented within the school context in reducing mental health problems in children, increasing their pro-environmental attitudes and behaviors as well as improving teachers’ well-being. Our intervention program will essentially entail 5-6^th^ grade teachers taking their students outside to a nearby green space (e.g. park, wooded area) during school hours for 2 h a week for 12 weeks. The teachers will be provided with a toolkit of outdoor lesson plans that were professionally designed to improve mental health and develop classic academic competencies.

Our main objective is to test the effectiveness of the *Open Sky School*, a nature-based school intervention, designed to decrease mental health problems in children, immediately after the intervention. We hypothesize that the *Open Sky School* will lead to greater reductions in overall mental health problems in children in comparison to a control group (primary outcome), in addition to greater improvements in children’s symptoms of depression, affect, connectedness to nature and pro-environmental behaviors and attitudes immediately after the intervention (secondary outcomes). We will explore whether the intervention improves teachers well-being and affect, and if benefits in primary and secondary outcomes are maintained at 3 months follow-up among children. We will also explore potential moderators in the association between exposure to the program and outcomes including the children’s sex [11, 13] and disability status [6], the quality of green-space surrounding school [14], the deprivation indicator of the school [12] and the amount of experience of teachers [15].

## Method and design

### Participants

#### Eligibility criteria

Participating schools will be recruited from a list of 280 French language elementary schools in the Canadian province of Quebec, enrolled in a larger study conducted by the Observatory for Children’s Education and Health, which aims to document the consequences of the COVID-19 pandemic on 4^th^ grade children’s academic achievement (www.observatoireenfants.ca/en). Additional inclusion criteria for schools are: (a) approval by their school board, (b) access to a natural environment (e.g., park, wooden area) on school grounds or within 1 km of the school and (c) having 5-6^th^ grade teachers provide informed and written consent to participate in this study (see Appendix 1). Inclusion criteria for children to participate in assessments are (a) enrollment in 5-6^th^ grade, (b) providing their assent (see Appendix 2) and (c) having their parents or legal guardians provide informed written consent (see Appendix 3). As indicated on the information and consent form, participants can withdraw from the study at any time.

### Intervention group

The intervention aims to provide approximately 2 h of exposure to nature per week and access to a toolkit of pedagogical and mental health activities grounded in positive psychology. The rationale for *Open Sky School* was informed by the growing literature on the benefits of spending time in nature for mental health [1]. The exposure to nature consists of teachers bringing their students to the highest quality green space within 1 km of a school which could be located on or off campus. For instance, this could be a forest on campus or a park nearby. The class will spend a total of 2 h (i.e. 2 one hour visits or one 2 h visits) per week for 12 weeks (transportation included), which is in line with recent guidelines recommending a minimum of 2 h per week in nature [16]. As summarized in Table 1, the main component of the *Open Sky School* consists of exposing children to nature, which is embedded in various activities (academic and mental health components), while providing teacher training and support. To this end, we designed an online toolkit of pedagogical and mental health activities. The mental health activities are rooted in positive psychology (mindfulness, philosophy for children and art therapy) and are designed to improve children’s mental health [17]. The pedagogical activities (French language, mathematics and sciences) are aligned on the core academic learning competences required by the Ministry of Education of Québec. Teachers will be allowed to use their own pedagogical activities (if they do, they will be asked to describe their activities), but are encouraged to use those provided in the toolkit, because they were professionally designed and positively appraised in the quality assessment phase. Nevertheless, as our trial primarily aims to reduce mental health problems among children, teachers will be required to carry out at least 10 mental health activities provided in the toolkit. The toolkit also includes brief video-based modules on best practices for implementing mental health activities outdoors (i.e., how to deal with negative emotions; how children can remain mindful in a noisy environment). Our experts and graduate students in education and clinical psychology will provide teachers with up to 1 h per week of virtual optional consultation during the intervention to discuss any arising issues related to the intervention and its implementation. Licensed psychologists from the research team will offer psychological support to any participant who reports high levels of psychological distress and orientate them to appropriate services if needed. More information about the *Open Sky School* can be found on the official website https://www.ecoleacielouvert.ca/ accessible to participants.

**Table 1:** Description of components within the intervention

Component	Description
Expose children to nature	Children and teachers are in contact with a natural environment (park or wooden area) situated > 1 km from school grounds or on school grounds *Duration*: 2 h per week for 12 weeks
Engage in activities	Teachers choose from an online toolkit of 30 mental health and pedagogical activities Teachers are required to choose a minimum of 10 mental health activities over the course of the intervention *Duration*: variable
Train and support teachers	Online video modules to train teachers in outdoor education and activities related to mental health *Duration*: at teacher’s discretion Virtual consultations between teachers and experts *Duration:* up to 1 h of support weekly

From May to June 2022, 34 elementary school teachers (5th and 6th grade) were invited to evaluate the quality of the 30 activities from our toolkit by answering 7 items about our activities (e.g. overall my students were engaged in the activity; I would use this activity with another class in the future) using a scale ranging from 1 (strongly disagree) to 5 (strongly agreed). Teachers were also invited to provide suggestions to improve the activity using an open response format. We received 85 questionnaires from 24 teachers reporting on 27 activities (14 mental health and 13 pedagogical). Teachers’ feedback was very positive with average ratings ranging from 4.2/5 to 4.5/5. More specifically, teachers responded that they agreed that the instructions clear and that the activities were simple to prepare and to implement. They reported that the students were engaged and liked the activity. Teachers agreed that they would use the activity in the future and recommend it to a colleague. Our research team further refined activities to incorporate teacher’s feedback.

### Control group

Six months after the inception of the trial, elementary schools (and their teachers) in the control condition will receive an unguided version of the intervention, supplemented by an online peer support group. As the children of the control group will by then be in high school with different teachers, we will provide them with a toolkit of 10 mental health activities that they can practice alone, in addition of support via video-conference, phone or email if they require help practicing the activities from a member of the research team. As in the intervention group, we will provide support by licensed psychologists to any children who report high levels of psychological distress and orient children to appropriate services if needed.

## Measures

Table **2** summarizes when and to whom each measure will be administered. Questionnaires will be administered before and after the intervention on campus in online format by trained research assistants or the classroom teacher. At follow-up, children will complete questionnaires online at home. We estimate that teachers will take 90 to 120 min to complete questionnaires (depending on the number of students in their classroom) and that students will take between 20 and 30 min to respond.

**Table 2:** Summary of measurements by informant and time points

			**Timeline**		
**Construct**	**Measure**	**Informant**	**T1**	**T2**	**T3**
**For children**					
***Mental health***					
Overall mental health symptoms	Social Behavior Questionnaire	Child	•	•	•
Overall mental health symptoms	Social Behavior Questionnaire	Teacher	•	•	
Depressive symptoms	Children’s Depression Inventory-Short Version	Child	•	•	•
Positive and negative affect	Positive and Negative Affect Schedule for Children	Child	•	•	•
***Relationship with environment***					
Connection with nature	Nature Connection Index	Child	•	•	•
Pro-environmental behaviors	Adapted from Keith et al. & Hickman et al	Child	•	•	•
***Appraisal of program***					
Appreciation of program ^a^	Ad hoc	Child		•	
**For teachers**					
Wellbeing	World Health Organization-5	Teacher	•	•	
Teacher enjoyment of outdoor education ^a^	Enjoyment of Teaching Mathematics Scale—adapted	Teacher		•	
Positive and negative affect	Positive and Negative Affect Schedule	Teacher	•	•	
**Moderators**					
Teacher qualifications and experience	Ad hoc	Teacher	•		
Teacher experience with outdoor education	Drawn from Ayotte-Beaudet et al	Teacher	•		
Green space of neighborhoods	Normalized Difference Vegetation Index	Linkage	•		
Disability status of children	Diagnosis of handicap, adaptation and learning disorders	Linkage	•		
Sex	Boy, girl	Linkage	•		
Deprivation indicator of the school	Ministry of education	Linkage	•		

*Note*. ^a^ measure administered in the intervention group only

Timeline: T1 = Baseline assessment (March 6 to 10 2023); T2 = 3 month assessment (June 5 to 12 2023); T3 = 6 month assessment (September 18 to 23 2023). Intervention takes place March 13 2023 to June 2 2023

### Primary outcome: Mental health symptoms

The Social Behavior Questionnaire [18] is a 30 item questionnaire that will be used to assess a range of mental health symptoms in children. The instrument incorporates items adapted from the Child Behavior Checklist [19], the Ontario Child Health Study Scales [20] and the Preschool Behavior Questionnaire [21] used in the Quebec Longitudinal Study of Children Development [22]. The frequency of children’s symptoms over the last 2 months is rated on a scale 3-point (never/not true = 0, sometimes/somewhat true = 1, often/very true = 2). Overall symptoms will be examined as outcomes, as well as internalizing symptoms (emotional distress and withdrawal; 11 items), externalizing symptoms (impulsive/hyperactive/inattentive and disruptive behaviors; 13 items), and social behaviors (pro-social behavior and peer relationships; 6 items) [23]. See Appendix 4 for all items in the questionnaire. Ratings will be obtained by both child and teacher reports which will be analyzed separately.

### Secondary outcomes

#### Additional mental health indicators for children

*The Positive and Negative Affect Schedule for Child (PANAS-C)* [24] is a 20-item scale that will be used to assess positive (e.g., “excited”) and negative (e.g., “upset”) affect which has good convergent and discriminate validity among children. Children will indicate to what extent they experience feelings over the last 2 weeks (1 = very slightly or not at all; 5 = extremely).

The *Children’s Depression Inventory-Short Version* (CDI-S) [25] includes 13 items that will be used to assess cognitive, affective and behavioral signs of depression in children. The CDI-S has good convergent, discriminate and factorial validity among children. Children consider how they were feeling over the last 2 weeks and respond on a 3-point scale (e.g. 1 = I hate myself; 2 = I don’t like myself; 3 = I like myself).

#### Relationship with environment for children

The *Nature Connection Index (NCI)* [26] is a self-report questionnaire including 6 items that will be used to assess connectedness to nature. The scale has good validity and reliability among children. Participants respond to affirmations (e.g. nature always makes me happy) using a 7-point scale (1 = strongly agree; 7 = strongly disagree).

*Pro-environmental attitude and behaviors* will be measured by a brief 6-item questionnaire developed in a recent study [27], that asks children to report the frequency they made an effort to conserve water, energy and recycle trash (1 = always; 5 = never) and the extent to which they agree that they are ready to volunteer, give money and talk to their entourage to protect nature (1 = strongly agree; 4 = strongly disagree). Additionally children will be asked about their pro-environmental behaviors in the context of climate change using 4 self-report questions, adapted from a previous study and refined by experts [9, 28]. Children report the extent to which they are worried about climate change (1 = not at all worried; 4 = extremely worried), that these worries motivate them to engage in pro-environmental behaviors or activism (1 = not at all; 4 = a lot) and that they feel capable of making behavioral changes to help the environment (1 = strongly disagree; 4 = strongly agree).

#### Well-being for teachers

The *World Health Organization Well-Being Index* (WHO-5) [29] is a 5-item self-report measure that will assess of wellbeing for adults. The WHO-5 is a widely used, valid and reliable measure that is sensitive to change. Participants indicate the frequency of their feelings (e.g. in a good mood) over the last two weeks (5 = all the time; 0 = never).

*The Positive and Negative Affect Schedule* (PANAS) [30] is a 20-item self-report measure that will be used to assess positive (e.g., “excited”) and negative (e.g., “upset”) affect which has well-established good psychometric properties. Teachers will indicate to what extent they experience feelings over the last two weeks (1 = very slightly or not at all; 5 = extremely).

### Appreciation of outdoor activities for children and teachers

An adapted version of the *Enjoyment of Teaching Mathematics Scale* [31] will be used for teachers from the intervention group at post-test to self-report their enjoyment of teaching outdoors (e.g. I really like teaching outdoors) with 5 items on a 5 point scale (1 = strongly agree; 5 = strongly disagree). We designed one item for children to self-report the frequency that they appreciated the intervention (1 = not at all; 4 = always) that will be administered in the intervention group at post-test.

### Moderator variables

#### Sex

The children’s sex (male, female) will be obtained via school records.

#### Disability status of children

Children formally diagnosed as having a physical or mental disability (e.g. intellectual deficiency) or having adaptation disorders (e.g. conduct disorders) or learning disorders (e.g. language deficits) will be identified via their school records.

#### Green space of neighbourhoods

The *Normalized Difference Vegetation Index* (NDVI) will be used to quantify the density of green vegetation associated with the school’s zip code. The widely used NDVI is based on the land surface reflectance of colors which is drawn from satellite images of the earth’s surface. The index varies between + 1 and -1 with higher values indicating higher green vegetation density. We will use 2019 satellite images which are available via the Consortium CANUE [32].

#### Deprivation indicator of the school

School’s level of deprivation will be quantified using a ranking provided by the Ministry of Education and Higher Education. The ranking is based on a composite score incorporating the proportion of students within each school whose mother completed high-school and whose both parents are employed full time. Schools are classified on a scale ranging from 1 (lowest deprivation) to 10 (highest deprivation) and scores of 8 to 10 are considered as disadvantaged [33].

#### Experience with outdoor teaching of teachers

Teachers’ experience with outdoor teaching over the last 3 years (e.g. context, duration) (e.g. did you practice outdoor education in autumn 2022?) and outdoor activities practiced in their leisure time will be assessed with a 6 questions that were successfully used in a previous study on school based outdoor education.

### Adherence assessments: Teacher Logbook

The teachers will fill out an online logbook in which they will indicate information about each nature visit. They will provide information on the activities they chose to carry out (from our toolkit or others of their choice), the duration of these activities, where the activity was carried out and the total time spent outdoors. They will record if they watched our training videos, if they encountered any problems and the amount of time they spent with our supervisors. This information will be used to evaluate the adequacy of the implementation of our intervention. To fully comply with implementation, there must be of a total of 24 h of intervention (2 h per week for 12 weeks) and a minimum of 10 mental health activities from the toolkit must be carried out. Classes that do not achieve a minimum of 80% completion and comply with content 90% of the time will be excluded from sensitivity analyses. Information contained in the logbooks will be evaluated independently by two researchers and we will analyze inter-rater agreement to ensure the consistency of the codification of the information provided.

### Sample Size

To determine sample size for primary outcome immediately after intervention, we conducted power analyses using a Monte Carlo simulation procedure (5000 samples). On the basis of the limited literature on outdoor education [11, 12], we expect to obtain a small effect size for our primary outcome, i.e. overall mental health problems in children. With 100 classes (n≈2500) which include typically 25 students (range between 18 to 26) per class, a refusal and attrition rate of 20% (n≈2,000) pre to post-test, an inter-class correlation of 0.02, an expected effect size of Cohen’s *d* = 0.20 and alpha set at 0.05, we expect that sufficient power will be achieved (> 0.80) for our primary and secondary outcomes.

### Randomization

Randomization will occur after baseline assessments. Once randomization has occurred, it is not possible to blind researchers and participants within our design. A simple computer algorithm will be used to randomly allocate schools to two parallel and balanced conditions (intervention or control; 1:1) by an independent researcher not involved in the study. This will ensure that all schools have an equal opportunity to be allocated into one of the two groups. In the event that multiple teachers from the same school consent to participate, they therefore be allocated to the same experimental condition to avoid contamination effects. All research assistants or classroom teachers administering questionnaires will complete training sessions prior to assessment to maintain consistency and when possible, the same investigator or classroom teacher will be used at baseline and at the immediate post-intervention assessments. The evaluation at 3 months follow-up will be completed by children online at home.

### Statistical analyses

All analyzes will be done at the cluster, i.e., school, level. Dependent variables will be measured on a continuous scale. A longitudinal ANCOVA, as described in Liu et al. (2009), will be used to estimate the main effects and the moderating effects previously mentioned. Participant characteristics from the intervention and control groups will be compared at the school level, where means (e.g., school deprivation indicator) could be added to the model as covariates in the unlikely event that randomization has not unable to balance the two groups. Multiple imputation and intent to treat analyses will be used to handle missing data.

### Recruitment strategy and timeline

**Fig. 1:**
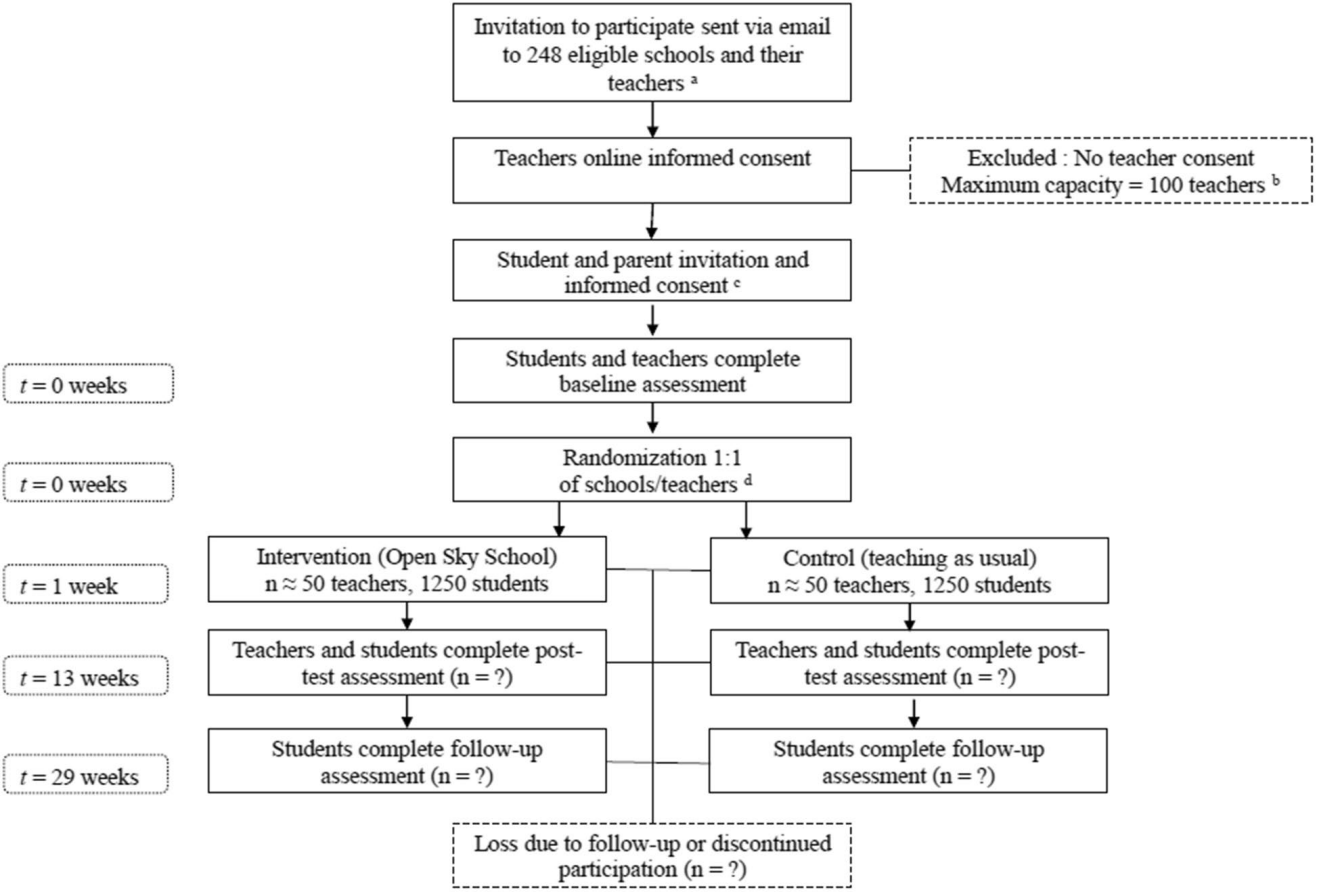
Flow chart of inclusion of participants *Note.* ^a^ 280 schools in 29 school boards were initially eligible. 4 school boards encompassing 32 schools did not agree to participate and were therefore removed from the recruitment process. ^b^ If more than 100 teachers consent to participate, we will randomly select 100 teachers to participate in the trial. ^c^ Parents and students must provide informed consent for students to participate in the assessments. Students who do not participate in assessments will still participate in the Open Sky School program. ^d^ In the event that multiple teachers from the same school consent to participate, they will be allocated to the same experimental condition to avoid contamination effects

For the randomized controlled trial, eligible schools will receive an initial invitation letter by email, with a 90 s video (December 2022), which they will be asked to distribute to their 5-6^th^ grade teachers. In the invitation letter, 5-6^th^ grade teachers will be invited to attend an online information session describing their expected involvement in the *Open Sky School* project. All interested teachers will be sent an information and consent form. Once teachers will have consented to take part in the study, they will then distribute information and consent forms to students and their parents. Parents will be invited to an online information session describing the intervention. Baseline assessment will take place from March 6^th^ to 10^th^ 2023 and post-test assessments will be conducted from June 5^th^ to 12^th^ 2023. The weekly 2 h nature visits will take place from March 13^th^ 2023 to June 2^th^ 2023. The follow-up assessments will be administered from September 18^th^ to 23^rd^ 2023. To encourage participation throughout the study, teachers will be compensated 100$CA for evaluating mental health symptoms of their students before and after the intervention (up to 200$CA total). Children can choose to be entered into a lottery to win gift cards to a local bookstore. More specifically, we will draw two 50$CA gift cards per class for children who complete questionnaires at pre and post-test, and ten 100$CA gift cards for children who the complete follow assessment. Figure 1 shows a flow chart of the inclusion of participants.

### Data collection, analysis and dissemination

Data will be collected online using Qualtrics, a widely used software in analytics and research. Confidential data (name, zip code) will be uploaded to a private and secure computer and then periodically erased from the server. The data file that will be analyzed will only contain the anonymous (numeric) identifier and the answers to the questionnaires. The file linking the identifiers to the data file will be kept on an encrypted USB key, protected by a password and kept in a locked safe. The safe will be kept in Dr. Geoffroy's and research coordinator work office. Only Dr. Geoffroy and the coordinator will have access to it. When data collection is complete, the data will be downloaded to a private computer and then deleted from Qualtrics. Members of the research team responsible for analyzing data will only have access to this anonymous data set which will be used solely for the purpose of establishing and disseminating research findings. Results will be disseminated to researchers, stake-holders, policy-makers and participants via scientific publications, oral presentations, interviews, and vulgarizations. All communications related to the project will be made available publically on the official website https://www.ecoleacielouvert.ca/.

## Discussion

### Potential implications

This study will quantify the extent to which an educational approach involving contact with nature, which is gaining popularity in the educational community, reduces mental health problems among children, while identifying the context in which the intervention is more effective. The intervention has a high potential for uptake by schools considering its flexibility and engaging activities. If successful, the program will be scaled up and used to issue guidelines for promoting mental health and healthy lifestyles in schools.

## Supplementary Information

**Additional file 1.**

## Data Availability

Data sharing not applicable to our randomized controlled trial protocol as no datasets have been generated or analyzed as of date. Data from the pre-testing of activities is available upon reasonable request.
