# Supplementary material for: Protocol for the Open Sky School: a two-arm clustered randomized controlled trial to test the effectiveness of a nature-based intervention on mental health of elementary school children
Source: BMC Public Health. 2023 Feb 3;23:236. doi: 10.1186/s12889-023-15033-y (PMC9896439; doi:10.1186/s12889-023-15033-y)
Supplement: Supplementary file 1 — Additional file 1. [file 12889_2023_15033_MOESM1_ESM.docx]

**Appendix 1: Teacher consent**

**FORMULAIRE D’INFORMATION ET CONSENTEMENT**

**Titre du projet de recherche**: École à Ciel Ouvert

**Chercheuse en charge du projet**

**de recherche**: Marie-Claude Geoffroy, PhD.

**Co-chercheur(s)**: Sylvana Côté, Catherine Malboeuf-Hurtubise,

Jean-Philippe Ayotte-Beaudet, Tianna Loose, Nicholas Chadi, Lise Gauvin, Geneviève Lessard, Isabelle Ouellet-Morin

**Membre(s) de l’équipe de recherche**: Katia Dumont

**Nom d de l'organisme**

**subventionnaire :** Instituts de recherche en santé du Canada

Observatoire pour l’Éducation et la Santé des Enfants

**Numéro du protocole** : 2022-581

**1. Introduction**

Nous vous invitons à participer à un projet de recherche. Cependant, avant d'accepter de participer à ce projet et de signer le formulaire de consentement, veuillez prendre le temps de lire, comprendre et examiner attentivement les informations suivantes.

Ce formulaire peut contenir des mots que vous ne comprenez pas. Nous vous invitons à poser toutes les questions que vous pourriez avoir à la chercheuse responsable de ce projet ou à un membre de son personnel de recherche et à leur demander d'expliquer tout ce qui n'est pas clair.

**2. Nature et objectifs du projet de recherche**

La pandémie de COVID-19 a eu des répercussions négatives sur le bien-être de plusieurs enfants. Il est impératif de trouver des moyens efficaces pour soutenir la santé mentale des enfants et atténuer les effets négatifs de la pandémie. Plusieurs études scientifiques suggèrent que l'exposition à la nature et l'éducation en plein air pourraient améliorer la santé mentale des enfants.

Nous avons développé une intervention de 12 semaines débutant en mars 2023 et se terminant en juin 2023 qui consiste à passer deux heures par semaine en nature dans l’enceinte de l’école ou dans un parc voisin. Les enfants s'adonneront alors à des activités pédagogiques dans la nature visant l’enseignement des sciences, des mathématiques, du français, des arts, de la philosophie et de la pleine conscience. Par exemple, les enfants pourront pratiquer la pleine conscience dans la nature (c’est-à-dire, porter attention à ce qui se passe autour d’eux ou apprendre à reconnaître les différents types de nuages).

L'objectif de ce projet de recherche est de tester les bienfaits du temps passé en nature, à l’école sur la santé mentale (anxiété, inattention) et ainsi favoriser le bien-être de tous les enfants.

**3. Déroulement du projet de recherche et nature de votre participation**

Nous menons ce que nous appelons un essai contrôlé randomisé avec un groupe intervention et un groupe contrôle, qui est une stratégie très reconnue pour tester l'efficacité des interventions. Environ 80-100 enseignant.es et leurs élèves participeront à l’étude.

Si vous acceptez de participer au projet de recherche, vous devrez répondre à deux questionnaires (mars et juin) à propos de votre enseignement, votre bien-être et les comportements de chacun de vos élèves (90-120 minutes par questionnaire). Vous devrez également prévoir un temps commun avec vos élèves pour que ceux-ci répondent à un questionnaire portant sur leur comportement et leur bien-être en classe (mars et juin). Vous recevrez une compensation financière pour chacun des questionnaires complétés.

À la suite du premier questionnaire, vous serez assigné-e au hasard soit au groupe d’intervention, soit au groupe contrôle.

SI VOUS ÊTES ASSIGNÉ-ES AU GROUPE INTERVENTION, vous passerez 2h par semaine dans un espace naturel situé dans l’enceinte de l’école ou dans un parc à proximité, durant les 12 semaines d'intervention. Vous pourrez enseigner le contenu pédagogique de votre choix ou mettre en pratique les activités développées par nos expert.es en pédagogie et psychologie scolaire.

Vous aurez accès à un ensemble de 30 activités élaborées par des personnes expertes en éducation et psychologie scolaire. Les activités seront présentées sur un site internet facile d’accès. Ces activités visent à développer un large éventail de compétences basées sur les compétences que doivent acquérir les élèves de 6e année primaire au Québec. Vous aurez le choix de réaliser les activités proposées dans la trousse d’activités fournie ou de créer vos propres activités pédagogiques, mais **vous devrez réaliser 10 activités de bien-être proposées dans la trousse d’activités**.

Chaque semaine, il vous sera demandé de répondre à quelques questions sur les activités que vous avez mises en œuvre, sur leur déroulement, et sur la formation et l’accompagnement proposé au moyen d’un journal de bord électronique.

SI VOUS ÊTES ASSIGNÉ.E AU GROUPE CONTRÔLE, vous devrez poursuivre votre enseignement comme à l’habitude pour l’année en cours, puis remplir le deuxième questionnaire avec vos élèves en juin. Vous réaliserez le programme d’intervention de septembre à décembre 2023.

Voici un tableau récapitulatif des tâches associées à chaque temps de mesure :

|  | **Mars 2023** | **Mars à Juin 2023 (12 semaines)** | **Juin 2023** |
| --- | --- | --- | --- |
| **Groupe intervention** | - Remplir questionnaire #1 - Prévoir un temps commun en classe (laboratoire informatique) pour que les enfants remplissent le questionnaire #1 (20 à 30 min) | - Préparation des activités (inclus dans votre préparation pédagogique) - Passer du temps dans un espace naturel dans l’enceinte de l’école ou à proximité et réaliser des activités pédagogiques, incluant au moins 10 activités visant le bien-être avec vos élèves - Remplir le journal de bord après chaque sortie (5 min) | - Remplir questionnaire #2 - Prévoir un temps commun en classe (laboratoire informatique) pour que les enfants remplissent le questionnaire #2 (20 à 30 min) |
| **Groupe contrôle** | - Remplir questionnaire #1 (enseignant-e, enfants) | Année scolaire typique ; réalisation de l’intervention à partir de septembre 2023 | - - Remplir questionnaire #2 (enseignant-e, enfants) |

*Nous pouvons fournir des questionnaires papiers aux enseignant.es qui le souhaitent.

**4. Inconvénients liés au projet de recherche**

L'équipe de recherche estime que la participation à ce projet de recherche ne comporte pas plus d’inconvénients que ceux habituellement reliés à votre travail ou au fait de se rendre dans un parc. Par exemple, :

- Inconfort dû aux allergies saisonnières
- Être responsable d’un groupe d’élèves à l’extérieur de l’enceinte de l’école
- Traverser la rue aux intersections de la circulation ;
- Blessures éventuelles dues à la marche ;
- Inconfort mineur lié aux conditions météorologiques saisonnières (pluie, vent, température, ...)
- Inconfort lors du remplissage des questionnaires
- Temps consacré à la recherche (questionnaires et journal de bord).

Enfin, si vous rencontrez des difficultés avec l'un des aspects de l'intervention, notre équipe de recherche est entièrement disponible pour vous fournir l’accompagnement et le soutien dont vous aurez besoin, à raison d’une heure par semaine.

**5. Risques associés au projet de recherche**

L'équipe de recherche estime que participer à ce projet de recherche comporte peu de risques pour vous.

**6. Avantages associés au projet de recherche**

Il se peut que vous retiriez un avantage personnel de votre participation à ce projet de recherche, mais nous ne pouvons pas vous l'assurer. La littérature suggère que de nombreuses personnes enseignantes aiment enseigner à l'extérieur et que passer du temps en nature est bénéfique pour la santé physique et mentale. En participant à ce projet, vous aurez accès à une trousse de 30 activités pédagogiques et de bien-être et nous vous fournirons un accompagnement lorsque vous les mettrez en œuvre.

Les élèves de votre classe peuvent bénéficier également de leur participation à ce projet de recherche. L'intervention est conçue pour améliorer la santé mentale et la connexion à la nature. De plus, nous espérons que les résultats obtenus contribueront à l'avancement des connaissances scientifiques dans ce domaine de recherche. Les résultats de cette étude pourraient profiter aux enfants du Québec et du monde entier. En effet, si l'intervention est efficace, elle pourrait être mise en œuvre dans un plus grand nombre d'écoles.

**7. Participation volontaire et possibilité de retrait**

Votre participation à ce projet de recherche est volontaire. Vous êtes donc libre de refuser de participer. Vous pouvez également vous retirer de ce projet à tout moment, sans avoir à fournir de raisons, en informant l'équipe de recherche.

Votre décision de ne pas participer à ce projet de recherche ou de vous en retirer n’aura aucune conséquence pour vous.

La chercheuse responsable du projet de recherche ou le comité d'éthique de la recherche du CIUSSS de l’Ouest-de-l’Île-de-Montréal peuvent mettre fin à votre participation, sans votre consentement. Cela peut se produire si de nouvelles découvertes ou informations indiquent que votre participation au projet n'est plus dans votre intérêt, si vous ne suivez pas les instructions du projet de recherche ou s'il existe des raisons administratives pour abandonner le projet.

Si vous vous retirez du projet ou si vous êtes retiré-e du projet, les informations déjà recueillies dans le cadre de ce projet seront néanmoins conservées, analysées ou utilisées pour assurer l'intégrité du projet.

Toute nouvelle connaissance acquise au cours du projet qui pourrait avoir un impact sur votre décision de poursuivre votre participation à ce projet vous sera communiquée rapidement.

**8. Confidentialité**

Durant votre participation à ce projet de recherche, la chercheuse responsable ainsi que les membres de son équipe de recherche rassembleront, dans un dossier de recherche, les renseignements vous concernant, nécessaires pour répondre aux objectifs scientifiques du projet de recherche.

Toutes les informations recueillies resteront confidentielles dans les limites autorisées par la loi. Afin de préserver votre identité et la confidentialité de ces informations, vous ne serez identifié.es que par un numéro d'identification de recherche. La clé du code reliant votre nom à votre dossier de recherche sera conservée par la chercheuse responsable et par la coordonnatrice du projet de recherche, sur une clé USB, cryptée, protégée par mot de passe et entreposée dans un coffre fermé à clé appartenant à la Dre Geoffroy.

Seules les personnes chercheuses et les membres de l’équipe de recherche auront accès aux données, préalablement anonymisées, pour des fins d’analyse.

Les informations recueillies en tant que données de recherche seront utilisées par la chercheuse responsable du projet et son équipe pour atteindre les objectifs scientifiques du projet décrits dans le présent formulaire d'information et de consentement.

Ces données de recherche seront conservées pendant au moins 7 ans après la fin de l'étude par la chercheuse responsable sur un serveur crypté, sécurisé de l’Université McGill, accessible à partir d’un ordinateur protégé par mot de passe. À l’issue de cette période, toutes les données seront effacées grâce à un logiciel de suppression des données (par exemple : Eraser). Les données de recherche pourront être publiées ou faire l'objet de discussions scientifiques, mais vous ne serez en aucun cas identifiable.

À des fins de surveillance, de contrôle, de protection, de sécurité, votre dossier de recherche sera accessible par une personne mandatée par les organismes de réglementation, par l'établissement ou par le comité d'éthique du CIUSSS de l’Ouest-de-l’Île-de Montréal.

**9. Possibilité de commercialisation**

 Les résultats de la recherche résultant de votre participation peuvent conduire à la création de produits commerciaux. Toutefois, vous ne recevrez aucun avantage financier.

**10. Financement du projet de recherche**

La chercheuse responsable de ce projet de recherche a reçu un financement des Instituts de recherche en santé du Canada pour mener à bien ce projet de recherche.

**11. Compensation**

En guise de compensation pour le temps passé à remplir les questionnaires associés au projet de recherche, vous recevrez un montant de 100 $ sous forme de carte-cadeau pour chacun des questionnaires, soit un montant total de 200 $ pour les 2 questionnaires. Si vous vous retirez du projet (ou si nous mettons fin à votre participation) avant qu’il ne soit complété, la compensation sera proportionnelle à la durée de votre participation.

**12. En cas de préjudice**

Si vous deviez subir quelque préjudice lié à ce projet de recherche, vous recevriez tous les soins et services requis par votre état de santé.

**13. Clause de responsabilité**

En acceptant de participer à ce projet de recherche, vous ne renoncez à aucun de vos droits et vous ne libérez pas la chercheuse responsable du projet de recherche, l’organisme subventionnaire et l’établissement de leur responsabilité civile et professionnelle.

**14. Identification des personnes-ressources**

Si vous avez des questions, si vous rencontrez des problèmes liés au projet de recherche ou si vous voulez retirer votre participation, vous pouvez contacter la chercheuse responsable du projet de recherche, le Dr Marie-Claude Geoffroy, au numéro de téléphone suivant : 514.761.6131, poste 4524 ou à l'adresse électronique : [ecoleacielouvert.recherche@gmail.com](mailto:ecoleacielouvert.recherche@gmail.com).

**15. Plaintes**

Pour toute question concernant vos droits en tant que participant-e à ce projet de recherche ou si vous avez des plaintes ou des commentaires à formuler, vous pouvez communiquer avec le commissaire aux plaintes et à la qualité des services du CIUSSS de l’Ouest-de-l’Île-de-Montréal, au 1 844 630-5125 ou par courriel à [commissariat.plaintes.comtl@ssss.gouv.qc.ca](mailto:commissariat.plaintes.comtl@ssss.gouv.qc.ca).

**16.** **Conflits d’intérêts**

La chercheuse principale déclare qu'elle n'a aucun intérêt personnel qui pourrait entrer en conflit avec son rôle de chercheuse.

**17. Suivi des aspects éthiques du projet de recherche**

Le comité d'éthique de la recherche du CIUSSS de l’Ouest-de-l’Île-de Montréal a approuvé le projet de recherche et en assure le suivi.

**Consentements spécifiques :**

- **Contacts ultérieurs pour participer à d'autres études**

Acceptez-vous d'être contacté à l'avenir pour être invité-e à participer à d'autres projets de recherche? Ces projets de recherche vous seront alors présentés et un consentement libre et éclairé sera demandé pour chaque projet. Seuls les projets ayant obtenu l'approbation éthique vous seront présentés.

**** **Oui** **** **Non**

Indiquez vos coordonnées pour être recontacté-e :

Nom : _______________________

Prénom : _______________________

Courriel : _______________________

- **Projets de recherche futurs (usage secondaire)**

Acceptez-vous que vos données de recherche soient utilisées par la chercheuse responsable du projet de recherche pour mener d'autres projets de recherche dans le même domaine de recherche? Cette utilisation secondaire sera conforme aux conditions d'utilisation des données mentionnées dans le formulaire de consentement éclairé, y compris la section 8 sur les exigences en matière de confidentialité et de date de destruction. Seuls les projets de recherche ayant obtenu une approbation éthique seront autorisés à utiliser vos données pour une utilisation secondaire.

**** **Oui** **** **Non**

**Déclaration de consentement**

J’ai pris connaissance du formulaire d’information et de consentement. Le projet de recherche et le présent formulaire d’information et de consentement m’ont été expliqué. J’ai pu obtenir les réponses à mes questions et on m’a laissé le temps voulu pour prendre une décision. Après réflexion,

**** je consens à participer au projet de recherche École à Ciel Ouvert

**** je refuse de participer au projet de recherche École à Ciel Ouvert

aux conditions qui y sont énoncées.

Nom du participant Signature Date

**ENGAGEMENT DE LA PERSONNE QUI OBTIENT LE CONSENTEMENT**

Je certifie que nous avons expliqué à la personne participante le présent formulaire d’information et de consentement et que nous avons répondu à ses questions, le cas échéant. Je m’engage, avec l’équipe de recherche, à respecter ce qui a été convenu au formulaire d’information et de consentement et à en remettre une copie signée et datée à la personne participante.

Nom SIgnature de la personne qui obtient le consentement Date

**Appendix 2: Child assent**

**Formulaire d'assentiment**

**Titre du projet de recherche**: École à Ciel Ouvert

**Chercheur responsable du projet**

**de recherche**: Marie-Claude Geoffroy, PhD.

**Co-chercheur(s)**: Sylvana Côté, Catherine Malboeuf-Hurtubise,

Jean-Philippe Ayotte-Beaudet, Tianna Loose, Nicholas Chadi, Lise Gauvin, Geneviève Lessard, Isabelle Ouellet-Morin

**Membre(s) de l’équipe de recherche**: Katia Dumont

**Nom d de l'organisme**

**subventionnaire :** Instituts de recherche en santé du Canada

Observatoire pour l’Éducation et la Santé des Enfants

**Numéro du protocole**: 2022-581

**Introduction**

Avant d'accepter de participer à cette étude et de signer ce document, prends le temps de lire et de comprendre les informations suivantes. Ce document peut contenir des mots que tu ne comprends pas. Tu peux poser des questions en tout temps à un membre de l’équipe de recherche ou à ton enseignant.e.

**Pourquoi avez-vous besoin de moi?**

Nous t’invitons, avec la permission de tes parents, à participer à une recherche menée à l'Institut universitaire en santé mentale Douglas. Avec cette étude, nous voulons tester l’efficacité de l’éducation dans la nature sur ton niveau de bien-être.

**Qu'est-ce que je vais devoir faire?**

Si tu acceptes de participer, tu seras placé.e dans l’un de ces 2 groupes : soit le groupe d’intervention ou le groupe contrôle. Tu ne peux pas choisir ton groupe, ce sera fait au hasard.

Si tu te retrouves dans le groupe d’intervention, tu iras passer 2h par semaine dans un espace naturel situé dans l’enceinte de l’école ou dans un parc à proximité (du mois de mars au mois de juin) avec les autres élèves de ta classe, pour améliorer votre bien-être. À chaque semaine, vous ferez des activités, en nature, pendant les heures de classe, pendant 12 semaines.

Si tu te retrouves dans le groupe contrôle, à partir de septembre 2023 tu auras accès à plusieurs activités sur un site internet et tu pourras les pratiquer en plein air dans tes temps libres.

Peu importe le groupe où tu seras, on te demandera de répondre à 3 questionnaires sur ton bien-être, ton comportement et ton rapport à la nature. Tu en compléteras deux en classe en mars et en juin et un à la maison en septembre. Ça te prendra de 20 à 30 minutes à chaque fois pour les remplir. Ton enseignant.e va aussi remplir un questionnaire sur ton comportement.

**Est-ce qu’il y a des risques ou des inconvénients si je participe?**

Il est possible que tu trouves un peu long de remplir tous les questionnaires, ou que tu te sentes mal à l’aise. Si tu te sens inconfortable, tu peux en parler à ton enseignant.e ou à tes parents ou téléphoner au numéro qui se trouvera en bas des questionnaires. Il est également possible que tu ressentes un inconfort lié aux conditions météos (la pluie, le vent, le froid, etc.) ou à des allergies saisonnières. Tu devras aussi être prudent.e pour ne pas te blesser en marchant ou en traversant la rue.

**Quels sont les avantages de participer?**

Les activités sont conçues pour améliorer ton bien-être. Si les résultats de cette étude sont positifs, d’autres enfants du Québec et du monde entier pourront profiter de ces activités en nature durant leurs journées d’école.

**Est-ce que je suis obligé.e de participer?**

Tu es libre d'accepter ou de refuser de remplir les questionnaires. . Même si tu acceptes maintenant de participer, tu peux arrêter n’importe quand en le disant à un membre de l’équipe de recherche, à ton enseignant.e ou à tes parents. Tu n’auras jamais à expliquer cette décision. Il est possible qu’on mette fin à ta participation pour diverses raisons.

**Qui saura que j’ai participé?**

En dehors de l'équipe de recherche, de ton enseignant.e et de tes parents, personne d'autre ne saura que les informations recueillies dans cette étude proviennent de toi. Cela signifie que nous prendrons soin de garder confidentielles toutes les informations personnelles qui t’identifient : ton nom, ton âge, ton adresse, etc.. Ces informations et tes réponses seront conservées dans un endroit sûr et seront détruites 7 ans après la fin du projet.

**Vais-je recevoir quelque chose en échange?**

Pour te remercier de ta participation et d’avoir répondu aux deux premiers questionnaires, tu auras la chance de participer à un tirage, parmi les élèves de ta classe : 2 prix de 50 $ en carte-cadeau en librairie seront tirés. En remplissant le 3e questionnaire, tu participeras au tirage de 10 cartes-cadeau de 100$ à l’automne 2023.

**Puis-je parler à quelqu'un si j'ai des questions?**

Si tu as des questions, si tu souhaites obtenir plus d'informations sur cette étude ou si tu veux arrêter, tu peux contacter Dre Marie-Claude Geoffroy qui est la chercheuse responsable du projet de recherche, au numéro de téléphone suivant : 514-398-2817 ou à l'adresse électronique : [ecoleacielouvert@gmail.com](mailto:ecoleacielouvert.recherche@gmail.com).

| **Déclaration et assentiment**   - Je comprends ce que l'on attend de moi si je si je réponds aux questionnaires. - Je comprends que si je refuse, je n'aurai aucune conséquence. - Je comprends que si j'accepte, je peux éventuellement m'arrêter à tout moment, sans explications et sans conséquences. - J'ai eu la chance de poser des questions.   J'accepte de participer à l'activité décrite dans ce document |
| --- |
|  |
| Nom _____________________________________________________ |
|  |
| Signature________________________________ Date __________________  **ou**  Assentiment verbal  Oui ___ Non ___ |

| **Déclaration de la personne ayant obtenu le consentement** |
| --- |
| J'ai expliqué au participant les termes de ce formulaire d'informations et de consentement et j'ai répondu aux questions qu'il m'a posées. |
|  |
| Nom _____________________________________________________ |
|  |
| Signature________________________________ Date_____________________ |

**Appendix 3 : Parent consent**

**FORMULAIRE D’INFORMATION ET CONSENTEMENT**

**Titre du projet de recherche**: École à Ciel Ouvert

**Chercheuse responsable du projet**

**de recherche**: Marie-Claude Geoffroy, PhD.

**Co-chercheur(s)**: Sylvana Côté, Catherine Malboeuf-Hurtubise,

Jean-Philippe Ayotte-Beaudet, Tianna Loose, Nicholas Chadi, Lise, Gauvin, Geneviève Lessard, Isabelle Ouellet-Morin.

**Membre(s) de l’équipe de recherche**: Katia Dumont

**Nom de l'organisme**

**subventionnaire :** Instituts de recherche en santé du Canada, Observatoire pour l’Éducation et la Santé des Enfants

**Numéro du protocole**: 2022-581

**1. Introduction**

Nous invitons votre enfant à participer à un projet de recherche. Cependant, avant d'accepter de participer à ce projet et de signer le formulaire de consentement, veuillez prendre le temps de lire, comprendre et examiner attentivement les informations suivantes.

Ce formulaire peut contenir des mots que vous ne comprenez pas. Nous vous invitons à poser toutes les questions que vous pourriez avoir à la chercheuse responsable de ce projet ou à la coordonnatrice de recherche.

**2. Nature et objectifs du projet de recherche**

La pandémie de COVID-19 a eu des répercussions négatives sur le bien-être de plusieurs enfants. Il est impératif de trouver des moyens efficaces pour soutenir la santé mentale des enfants et atténuer les effets négatifs de la pandémie. Plusieurs études scientifiques suggèrent que l'exposition à la nature et l'éducation en plein air pourraient améliorer la santé mentale des enfants.

Nous avons développé une intervention de 12 semaines débutant en mars 2023 et se terminant en juin 2023 qui consiste à passer deux heures par semaine en nature dans l’enceinte de l’école ou dans un parc voisin. Les enfants s'adonneront alors à des activités pédagogiques dans la nature visant l’enseignement des sciences, des mathématiques, du français, des arts, de la philosophie et de la pleine conscience. Par exemple, les enfants pourront pratiquer la pleine conscience dans la nature (c.-à-d., porter attention à ce qui se passe autour d’eux) ou apprendre à reconnaître les différents types de nuages.

L'objectif de ce projet de recherche est de tester les bienfaits du temps passé en nature, à l’école sur la santé mentale (anxiété, inattention) et ainsi favoriser le bien-être de tous les enfants.

**3. Déroulement du projet de recherche**

**3.1 Lieu du projet de recherche, durée et nombre de visites**

Nous menons ce que nous appelons un essai contrôlé randomisé avec un groupe intervention et un groupe contrôle, qui est une stratégie très reconnue pour tester l'efficacité des interventions. Environ 2000 élèves et leurs enseignant.es participeront à l’étude.

Si votre enfant est assigné au **groupe intervention**, cela signifie qu’il passera 2h par semaine dans un espace naturel situé dans l’enceinte de l’école ou dans un parc à proximité. Cet espace naturel peut être situé dans l’enceinte de l’école (par exemple, dans un boisé) ou dans un parc situé à proximité de l’école (maximum d’un kilomètre) (par exemple, un parc urbain). Sur une période de 12 semaines, soit entre mars et juin 2023, les élèves seront exposés à des périodes d’apprentissage en nature pendant deux heures par semaine (une période de deux heures ou deux périodes d’une heure). Les enseigant.es pourront enseigner le contenu pédagogique de leur choix ou mettre en pratique les activités développées par nos expert.es en pédagogie et psychologie scolaire.

Si votre enfant est assigné au **groupe contrôle**, cela signifie qu’il ne recevra pas l’intervention, mais il aura accès (ainsi que les autres élèves de sa classe) à une banque de 10 activités en ligne, développées par des expert-es en éducation et en santé mentale, à compter de septembre 2023 (par exemple, méditation guidée et mandala en lien avec la nature).

Votre enfant sera assigné-e à un des deux groupes au hasard. Les deux groupes (intervention et contrôle) rempliront 3 questionnaires de 20 à 30 minutes, soit en mars, juin (en classe) et septembre 2023 (à la maison).

**3.2 Nature de la participation de votre enfant**

En plus de participer aux activités en nature (groupe d’intervention), ou d’avoir accès aux activités en ligne (groupe contrôle), votre enfant remplira des questionnaires sur son bien-être, son comportement et son rapport à la nature. Les questionnaires seront administrés trois fois et prendront entre 20 et 30 minutes à remplir. Les questionnaires seront d'abord administrés dans la classe au retour de la semaine de relâche, en mars 2023, puis la première semaine de juin 2023. Afin de tester les effets à moyen terme de l'intervention, nous demanderons à votre enfant de remplir à nouveau les questionnaires, en ligne, en septembre 2023.

Voici un tableau récapitulatif des tâches associées à chaque temps de mesure :

| **Mars 2023** | **Mars-Juin 2023 (12 semaines)** | **Juin 2023** | **Septembre 2023** |
| --- | --- | --- | --- |
| Remplir questionnaire #1 | *Groupe intervention* : passer du temps dans un espace naturel dans l’enceinte de l’école ou à proximité et participer à des activités pédagogiques, incluant au moins 10 activités visant le bien-être.  *Groupe contrôle* : année scolaire typique | Remplir questionnaire #2 | Remplir questionnaire #3 |

**4. Inconvénients associés au projet de recherche**

L'équipe de recherche estime que la participation à ce projet ne comporte pas plus d’inconvénients que ceux habituellement reliés au fait de se rendre dans un parc. Par exemple, au cours d'une activité extérieure typique, il y a des risques liés aux éléments suivants :

- Inconfort dû aux allergies saisonnières
- Traverser la rue aux intersections de la circulation;
- Blessures éventuelles dues à la marche ;
- Inconfort mineur lié aux conditions météorologiques saisonnières (pluie, vent, température, ...)
- Temps consacré aux questionnaires

Enfin, le fait de répondre à des questions sur ce qu'ils ressentent peut parfois mettre les enfants mal à l'aise. S'ils se sentent tristes ou s’ils ont des questions, ils peuvent parler à leur enseigant.e ou à un membre de l'équipe de recherche. N’hésitez pas à leur proposer de nous écrire si c’est le cas.

**5. Risques associés au projet de recherche**

L'équipe de recherche estime que participer à ce projet de recherche comporte peu de risques pour votre enfant.

**6. Avantages associés au projet de recherche**

Votre enfant peut retirer un avantage personnel de sa participation à ce projet de recherche, mais nous ne pouvons pas vous l'assurer. Les activités sont conçues pour améliorer la santé mentale et la connexion à la nature. Si votre enfant est assigné au groupe contrôle, nous lui donnerons accès à un site internet incluant 10 activités visant à promouvoir le bien-être et qu’il pourra réaliser par lui-même dès septembre 2023. De plus, les résultats de cette étude pourraient profiter aux enfants du Québec et du monde entier. Si le temps passé en nature à l’école est efficace, les résultats pourraient être mis en œuvre dans un plus grand nombre d'écoles.

**7. Participation volontaire et possibilité de retrait**

La participation de votre enfant à ce projet de recherche est volontaire. Vous êtes donc libre de refuser qu'il remplisse les questionnaires. Vous pouvez également le retirer de ce projet à tout moment, sans avoir à fournir de raisons, en informant l'équipe de recherche.

Votre décision de refuser que votre enfant participe à ce projet de recherche ou de le retirer n’aura aucune conséquence pour vous ou pour lui.

La chercheuse responsable du projet de recherche ou le comité d'éthique de la recherche du CIUSSS de l’Ouest-de-l’Île-de-Montréal peuvent mettre fin à la participation de votre enfant, sans votre consentement. Cela peut se produire si de nouvelles découvertes ou informations indiquent que la participation de votre enfant au projet n'est plus dans son intérêt, s'il ne suit pas les instructions du projet de recherche ou s'il existe des raisons administratives pour abandonner le projet.

Si vous retirez votre enfant du projet ou s’il est retiré du projet, les informations déjà recueillies dans le cadre de ce projet seront néanmoins conservées, analysées ou utilisées pour assurer l'intégrité du projet.

Toute nouvelle connaissance acquise au cours du projet qui pourrait avoir un impact sur votre décision de poursuivre la participation de votre enfant à ce projet vous sera communiquée rapidement.

À la fin de ce formulaire, il vous sera demandé si vous acceptez que votre enfant remplisse les questionnaires.

**8. Confidentialité**

Durant la participation à ce projet de recherche, la chercheuse responsable ainsi que les membres de son équipe de recherche rassembleront, dans un dossier de recherche, les informations concernant votre enfant, nécessaires pour atteindre les objectifs scientifiques de cette recherche.

Toutes les informations recueillies resteront confidentielles dans les limites autorisées par la loi. Afin de préserver l’identité des enfants et la confidentialité de ces informations, ils ne seront identifiés que par un numéro d'identification numérique de recherche. La clé du code reliant leur nom à leur dossier de recherche sera conservée par la chercheuse responsable et la coordonnatrice de ce projet de recherche, sur une clé USB cryptée, protégée par mot de passe et entreposée dans un coffre fermé à clé appartenant à la Dre Geoffroy.

Seuls les membres de l’équipe de recherche auront accès aux données, préalablement anonymisées, pour des fins d’analyse.

Les informations recueillies en tant que données de recherche seront utilisées par la chercheuse responsable du projet pour atteindre les objectifs scientifiques du projet décrits dans le présent formulaire d'information et de consentement.

Ces données de recherche seront conservées pendant au moins 7 ans après la fin de l'étude par la chercheuse responsable sur une plateforme sécurisée de l’Université McGill. À l’issue de cette période, toutes les données seront effacées grâce à un logiciel de suppression des données (par exemple Eraser). Les données de recherche pourront être publiées ou faire l'objet de discussions scientifiques, mais votre enfant ne sera en aucun cas identifiable.

À des fins de surveillance, de contrôle, de protection, de sécurité, le dossier de recherche de votre enfant sera accessible par une personne mandatée par les organismes de réglementation, par l'établissement ou par le comité d'éthique de la recherche du CIUSSS de l’Ouest-de-l’Île-de Montréal.

**9. Possibilité de commercialisation**

Les résultats de la recherche résultant de la participation de votre enfant pourraient conduire à la création de produits commerciaux. Toutefois, ni vous ni votre enfant ne recevrez d’avantages financiers.

**10. Financement du projet de recherche**

La chercheuse responsable de ce projet de recherche a reçu un financement des Instituts de recherche en santé du Canada et de l’Observatoire pour l’Éducation et la Santé des Enfants pour mener à bien ce projet de recherche.

**11. Compensation**

En guise de compensation pour leur participation au projet de recherche tous les enfants ayant répondu aux deux premiers questionnaires participeront au tirage de 2 prix de 50$ par classe participante sous forme de carte-cadeau, en librairie. Si vous retirez votre enfant du projet ou s’il a mis fin à sa participation avant que le projet ne soit complété à l’école (questionnaire #2 en juin 2023), il ne pourra pas participer au tirage. 10 prix de 100$ en carte-cadeau seront aussi tirés parmi tous les enfants qui répondront au questionnaire à la maison (questionnaire #3).

**12. En cas de préjudice**

Si votre enfant subit un quelconque préjudice du fait de sa participation au projet de recherche, il recevra tous les soins et services requis par son état de santé.

**13. Clause de responsabilité**

En acceptant de participer à ce projet, vous ne renoncez à aucun de vos droits ni ne libérez les chercheur.ses de leurs obligations civiles et professionnelles.

**14. Identification des personnes-ressources**

Si vous avez des questions, si vous rencontrez des problèmes liés au projet de recherche ou si vous voulez retirer la participation de votre enfant, vous pouvez contacter la chercheuse responsable du projet de recherche, la Dre Marie-Claude Geoffroy, à l'adresse électronique suivante : [ecoleacielouvert.recherche@gmail.com](mailto:ecoleacielouvert.recherche@gmail.com).

**15. Plaintes**

Pour toute question concernant vos droits ou ceux de votre enfant en tant que participant à ce projet de recherche ou si vous avez des plaintes ou des commentaires à formuler, vous pouvez communiquer avec le commissaire aux plaintes et à la qualité des services du CIUSSS de l’Ouest-de-l’Île-de-Montréal, au 1 844 630-5125 ou par courriel à [commissariat.plaintes.comtl@ssss.gouv.qc.ca](mailto:commissariat.plaintes.comtl@ssss.gouv.qc.ca).

**16. Conflits d'intérêts**

La chercheuse principale déclare qu'elle n'a aucun intérêt personnel qui pourrait entrer en conflit avec son rôle de chercheuse.

**17. Suivi des aspects éthiques du projet de recherche**

Le comité d'éthique de la recherche du CIUSSS de l’Ouest-de-l’Île-de Montréal a approuvé le projet de recherche et en assure le suivi.

**Consentements spécifiques:**

- **Contacts ultérieurs pour participer à d'autres études**

Acceptez-vous d'être contacté.e à l'avenir pour être invité.e à participer à d'autres projets de recherche? Ces autres projets de recherche vous seront alors présentés et un consentement libre et éclairé sera demandé pour chaque projet. Seuls les projets ayant obtenu l'approbation éthique vous seront présentés.

**  Oui** ** Non**

Indiquez vos coordonnées pour être recontacté-e :

Nom : _______________________

Prénom : _______________________

Courriel : _______________________

- **Projets de recherche futurs (usage secondaire)**

Acceptez-vous que les données de recherche de votre enfant soient utilisées par la chercheuse responsable du projet de recherche pour mener d'autres projets de recherche dans le même domaine? Cette utilisation secondaire sera conforme aux conditions d'utilisation des données mentionnées dans le formulaire de consentement éclairé, y compris la section 8 sur les exigences en matière de confidentialité et de date de destruction. Seuls les projets de recherche ayant obtenu une approbation éthique seront autorisés à utiliser vos données pour une utilisation secondaire.

** ** **Oui** ** ** **Non**

- **Utilisation d’informations complémentaires**

J’accorde aux personnes chercheuses le droit d’accéder et d’utiliser des informations sur mon enfant recueillies au préalable par l’Institut de la Statistique du Québec dans le cadre de l’Observatoire pour l’Éducation et la Santé des Enfants (par exemple, résultats sur l’Épreuve ministériel de français en 4e année primaire s’il y a lieu).

**** **Oui**  **** **Non**

J’accorde aux personnes chercheuses le droit de recueillir **ultérieurement** des informations complémentaires sur mon enfant auprès de ministères et d’organismes afin de réaliser des travaux statistiques liés à l’objectif général de l’étude, c’est-à-dire obtenir des données administratives ou d’enquêtes concernant mon enfant dans divers secteurs, notamment ceux :

- de l’éducation (p. ex. ministère de l’Éducation, centres de services scolaires)
- de la santé (p. ex. Régie de l’assurance maladie du Québec, ministère de la Santé et des Services sociaux, Registre de vaccination du Québec)

Les informations complémentaires recueillies seront couplées aux données de l’étude à l’aide d’un numéro d’identification anonyme, et seront accessibles uniquement dans un laboratoire sécurisé de l’Institut de la Statistique du Québec (CADRISQ, Centre d’accès aux données de recherche). Votre enfant ne pourra donc être identifié-e.

**** **Oui**  **** **Non**

J’accorde aux personnes chercheuses le droit de jumeler l’ensemble des renseignements recueillis sur mon enfant dans le cadre de l’étude, de façon dépersonnalisée (non identifiable), et ce strictement pour des fins statistiques et de recherche. Cette opération se fera uniquement pour réaliser des travaux en lien avec l’objectif général de l’étude et en utilisant l’environnement sécurisé de Statistique Québec.

**** **Oui**  **** **Non**

**Déclaration de consentement**

Acceptez-vous que l**’enseignant-e** de votre enfant remplisse les questionnaires au sujet de son de son comportement afin de vérifier l’efficacité d’École à Ciel Ouvert ?

**** **Oui**  **** **Non**

J’ai pris connaissance du formulaire d’information et de consentement. Le projet de recherche et le présent formulaire d’information et de consentement m’ont été expliqué. J’ai pu obtenir les réponses à mes questions et on m’a laissé le temps voulu pour prendre une décision libre et éclairée. Après réflexion,

**** j'accepte que mon enfant remplisse les questionnaires

**** je refuse que mon enfant remplisse les questionnaires

______________________________________________________________________________

Nom du parent Signature du parent /tuteur  Date

Adresse courriel du parent/tuteur pour envoi du questionnaire #3 : ____________________________________________________

Numéro de téléphone du parent/tuteur : _______________________

**ENGAGEMENT DE LA PERSONNE QUI OBTIENT LE CONSENTEMENT**

Je certifie que nous avons expliqué à la personne participante le présent formulaire d’information et de consentement et que nous avons répondu à ses questions, le cas échéant. Je m’engage, avec l’équipe de recherche, à respecter ce qui a été convenu au formulaire d’information et de consentement et à en remettre une copie signée et datée à la personne participante.

______________________________________________________________________________

Nom Signature de la personne qui obtient le consentement Date
